# Cardiomyopathies and Related Changes in Contractility of Human Heart Muscle

**DOI:** 10.3390/ijms19082234

**Published:** 2018-07-31

**Authors:** Petr G. Vikhorev, Natalia N. Vikhoreva

**Affiliations:** 1 National Heart and Lung Institute, Imperial College London, London W12 0NN, UK; 2 Heart Science Centre, Magdi Yacoub Institute, Harefield Hospital, London UB9 6JH, UK; nataliav@myi.org.uk

**Keywords:** human heart muscle contractility, dilated cardiomyopathy, hypertrophic cardiomyopathy, troponin phosphorylation, cardiomyopathy mutations, Ca^2+^-sensitivity, length dependent activation

## Abstract

About half of hypertrophic and dilated cardiomyopathies cases have been recognized as genetic diseases with mutations in sarcomeric proteins. The sarcomeric proteins are involved in cardiomyocyte contractility and its regulation, and play a structural role. Mutations in non-sarcomeric proteins may induce changes in cell signaling pathways that modify contractile response of heart muscle. These facts strongly suggest that contractile dysfunction plays a central role in initiation and progression of cardiomyopathies. In fact, abnormalities in contractile mechanics of myofibrils have been discovered. However, it has not been revealed how these mutations increase risk for cardiomyopathy and cause the disease. Much research has been done and still much is being done to understand how the mechanism works. Here, we review the facts of cardiac myofilament contractility in patients with cardiomyopathy and heart failure.

## 1. Introduction

The main function of the heart is pumping the blood around the body according to the organism’s physiological requirements [1]. Heart contraction is initiated by the action potential propagated from sinoatrial node pacemaker cells. Membrane depolarization opens voltage-gated L-type Ca^2+^ channels in the cytoplasmic membrane. The initial influx of Ca^2+^ further stimulates efflux of Ca^2+^ from sarcoplasmic reticulum via ryanodine receptor type-2 channels [2,3]. Ca^2+^ binds to troponin C of the troponin complex, which is composed of three subunits: troponin C, I, and T. Troponin is attached to tropomyosin, which lies along the actin filament, and regulates its position depending on the Ca^2+^ concentration [4,5]. Tropomyosin blocks myosin binding sites on actin at low Ca^2+^ concentration. When [Ca^2+^] increases, tropomyosin releases the sites and promotes interaction of myosin with actin. Uptake of Ca^2+^ from cytoplasm by sarcoplasmic/endoplasmic reticulum Ca^2+^-ATPase Na^+^/Ca^2+^ exchanger stimulates relaxation [2,3]. It is widely accepted that myofilament Ca^2+^ sensitivity is regulated via phosphorylation of troponin I (TnI) at Ser 23 and 24 [6,7,8]. The phosphorylation of other proteins, such as myosin binding protein C [9] and regulatory light chain of myosin, also can modulate Ca^2+^ sensitivity [10,11,12] but to a lesser degree.

Cardiomyopathy is a disease of the heart muscle leading to cardiac dysfunction with characteristic pathological remodeling and eventually to heart failure. Besides cardiomyopathies, heart failure can be caused by other conditions, particularly by coronary artery disease and hypertension which are the leading causes of cardiac death [13]. There are several different types of cardiomyopathies: hypertrophic cardiomyopathy (HCM), dilated cardiomyopathy (DCM), restrictive (RC), and arrhythmogenic right ventricular (ARVC) [14,15,16,17,18]. Hypertrophic cardiomyopathy, DCM, and ARVC have been recognized as genetic diseases [19,20].

Dilated cardiomyopathy and HCM are the most common types of cardiomyopathies and they have been intensively studied. Hypertrophic cardiomyopathy affects one in 500 of the population and DCM affects one in 250–500 [17]. They both have been implicated with a high risk of sudden cardiac death, where DCM accounts for 30–40% of heart failure [13,19,21]. Dilated cardiomyopathy and HCM decrease stroke volume and cardiac output but in different ways [22]. Hypertrophic cardiomyopathy is characterized by a pathologically hypertrophied (wall thickness > 15 mm) left ventricle and septum and without left ventricular cavity dilatation. This leads to a decrease of the intraventricular dimension and blood flow obstruction resulting in diastolic dysfunction. In about 10% of patients, HCM may progress further to a dilated phenotype [23]. The morphologic macroscopic changes do not always become apparent. Myocyte disarray exceeding 5–10% is the recognized trademark for HCM diagnosis [24]. On the contrary, the left ventricle of a DCM heart is dilated and the walls become thinner [25]. The heart cannot contract properly and left ventricular ejection fraction is reduced (<45%; systolic dysfunction). Hypertrophic cardiomyopathy and DCM can show morphological variations [23,25,26,27] and often can be misdiagnosed.

Dilated cardiomyopathy can be initiated by different conditions: autoimmune diseases (diabetes, thyroid disease), infections, and toxins (alcohol, chemotherapy), high blood pressure, heart attack or coronary artery disease [28,29]. It should be noted that the case of dilated cardiomyopathy which develops after a myocardial infarction or ischemia is often classified as a separate disease entity: ischemic cardiomyopathy (ICM) [25,28,29,30,31]. The environmental cases can also have genetic predisposition [32,33]. If known cause is not identified, DCM is called idiopathic DCM. Idiopathic DCM accounts for 50% of all cases [25,34,35] and its prevalence in children is higher (66%) [36]. Family screening shows that 30–50% of all idiopathic DCM cases are familial [19]. Whole genome sequencing analysis revealed that mutations were the main underlying cause of the disease in about 50% of patients diagnosed with idiopathic DCM. Dilated cardiomyopathy is a polygenetic disease with mutations found in sarcomeric, structural, and other protein genes; mutations in 57 genes have been linked to DCM [20]. Where mutations in the titin gene (*TTN)* are the most frequent (11.6–21.4%) [37,38,39] and account for ~25–27.6% of familial [37,40] and 11.6–18% of sporadic [37,38] genetic cases. The genes of sarcomeric proteins more frequently identified as genetic cause are *MYH7* (β-cardiac myosin heavy chain), *TPM1* (tropomyosin alpha-1 chain), and the genes of cardiac troponins *TNNT2* (troponin T), *TNNI*3 (troponin I), and *TNNC1* (troponin C).

In the case of HCM, pathogenic mutations, which were found in 37.9–63.2% cases [41,42,43,44,45,46,47], are located mostly in the genes of nine sarcomeric proteins: *MYH7*, *MYBPC3* (cardiac myosin binding protein C), *TNNT2*, *TNNI3*, *TNNC1*, *TPM1*, *ACTC* (cardiac actin), *MYL2* (ventricular myosin light chain 2, LC2), and *MYL3* (myosin light chain 3) [42,43,48,49,50]. Where 74–85.1% of all mutations are in the genes of *MYBPC3* (36.2–54.8%) and *MYH7* (25–48.9%) [42,43,44,45,46,47].

In our review we summarize the changes of contractile property of human cardiac muscle associated with DCM and HCM.

## 2. Approaches and Parameters to Estimate Contractility

It is recognized that myofibril contractile dysfunction plays a central role in initiation and progression of cardiac disease. However, how pathogenic mutations increase risk of cardiomyopathies or cause the diseases is unclear. The common explanation is that mutations in the contractile and regulatory proteins of sarcomere disturb muscle contraction. Mutations in titin change viscoelasticity properties, and mutations in other non-contractile proteins may induce defects in cell signaling pathways that modify cardiac response. But it seems that the mechanism is complex and there is no model that explains the mechanism of disease.

Numerous studies have been done using patient heart samples as well as animal models of cardiomyopathies and chimeric protein constructs with recombinant proteins from different sources to understand the diseases. However, in this review we would like to summarize the experimental data obtained only from human heart muscle samples. Skinned muscle strips, isolated cardiomyocytes, and myofibrils obtained from frozen patient hearts [51] were used to study heart contractility [52,53,54]. As DCM and HCM affect the left ventricle more, most of the research has been done on left ventricular and septum samples, and not much is known about whether there are significant abnormalities in the contractility of the atriums and right ventricle.

The fundamental parameters used to describe muscle contractility are: the force generating capacity (*F*_max_) and Ca^2+^-sensitivity (*EC*_50_; and *n*_H_ is a parameter characterizing the steepness of the sigmoidal curve). Furthermore, the fast kinetics of myofilament contractility can be measured only in a single myofibril, due to the relatively slow diffusion in samples more than a few microns thick, such as cardiomyocytes. This technique is far more advantageous in respect to the understanding of the molecular mechanisms of cardiomyopathies as it also provides us with data on myosin cross-bridge kinetics and the mechanisms of activation and relaxation in a single muscle myofibril [53,54]. Once [Ca^2+^] reaches a certain threshold, force rapidly develops until it reaches plateau (*F*_max_). The time course of force development can be fitted with a single exponential function characterized by the rate constant *k*_ACT_. The activation rate is highly dependent on the concentration of Ca^2+^ in activating solution.

Relaxation has two phases: slow and fast. The first phase is nearly isotonic, with a slow linear decline in tension, and it lasts about 100 ms. The slow phase can be fit with a linear function and characterized by the rate constant *k*_LIN_, which is calculated from the slope value of the linear fit by normalizing it to the maximum tension ($k_{\mathrm{LIN}}\;=\;-\mathrm{slope}/F_{\max}$), and the duration time *t*_LIN_. After the latent period, the slow phase translates into an exponential force decay phase. The rate constants of the exponential force development (*k*_ACT_) and the fast phase of relaxation (*k*_REL_) are fitted with a single exponential function ($\;y\left(t\right)=y_{\mathrm{plateau}}+\left(y_{0}-y_{\mathrm{plateau}}\right)\exp\left(-kt\right)$, where the parameter *k*, (*k*_ACT_, *k*_TR_ or *k*_REL_) characterize the rate of the course [55]. In order to determine the rate of myofibril activation in thick specimens, such as cardiomyocytes, a fast length release following quick restretch is applied to cardiac cells or muscle strips and the rate of force redevelopment (*k*_TR_) that is comparable to *k*_ACT_ is measured [56].

## 3. Contractile Properties of HCM and DCM Hearts

### 3.1. Decreased Force Capacity

**Figure 1:**
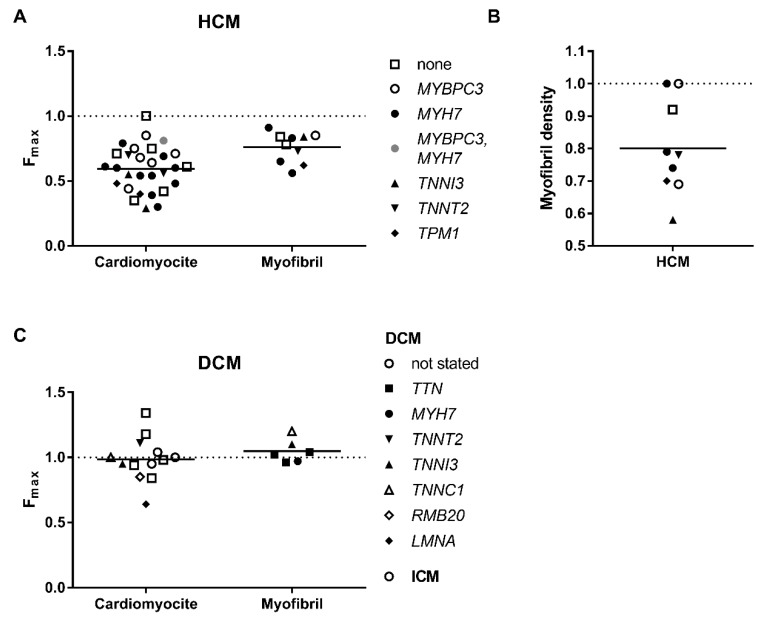
Maximum force of contraction by different types of preparations: from hearts of patients with HCM (**A**) and DCM (**C**). As can be seen, the maximal force generated by cardiomyocytes as well as myofibrils is lower in the HCM samples. The force is not diminished in DCM samples. (**B**) Density of myofibrils in cells were measured in some HCM samples. Each data point represents a different experimental group where the symbols indicate genes where mutations were found. See also Table 1 and Table 2. All values are normalised to those of donor heart muscle (dashed line).

Hypertrophic cardiomyopathy samples produce significantly lower strength per muscle cross-sectional areas (Figure 1A). The decrease in force in muscle tissue and isolated cardiomyocytes can be partially explained by myofibril disarray [14] and decrease of myofibril density (Figure 1B), but also due to the contractile defects. In fact, the maximal force developed by cardiomyocytes from HCM hearts was lower almost in all studied cases (Figure 1A, Table 1). The mean *F*_max_ was 41% lower than that of healthy donor hearts. At the same time, myofibril density was 20% lower in HCM cardiomyocytes [14,57,58,59]. Furthermore, the force produced by single myofibrils from HCM hearts with an identified genetic disease-causing mutation, irrespective of gene (*MYBPC3*, *MYH7*, *TPM1*, *TNNT2*), was also lower by 24% than that of control heart myofibrils (Figure 1A). This can suggest that there is some abnormality in cross-bridge cycling kinetics. However, the effect does not have to be purely due to mutation. Other factors as haploinsufficiency and secondary changes such as an altered protein phosphorylation [60], protein oxidation [61,62,63], and changes in protein isoform expression (see review [64]) cannot be excluded. Indeed, a number of samples with no mutation found in the genes of sarcomeric proteins also showed significantly lower (by 22%) myofibrillar force [57].

With respect to DCM samples, neither cardiomyocytes nor myofibrils produce force significantly different from that of donor hearts, except in one study with mutation in *LMNA* (lamin A/C; p.(R331Q)) [78,79] (Figure 1C, Table 2), where *F*_max_ was lower by 37% and that was explained by the decrease in myofibril density (Table 2). This means that systolic dysfunction observed in DCM hearts is not caused by the decrease in force production, but is rather due to ventricular dilation. The end-diastolic volume increase causes a decrease in left ventricular ejection fraction [91]. A decrease in myofibril density was also observed in the case of end-stage pediatric cardiomyopathy [58] and congenital dilated cardiomyopathy with mutation in the structural protein filamin-C [92].

### 3.2. Activation and Relaxation Kinetics

**Figure 2:**
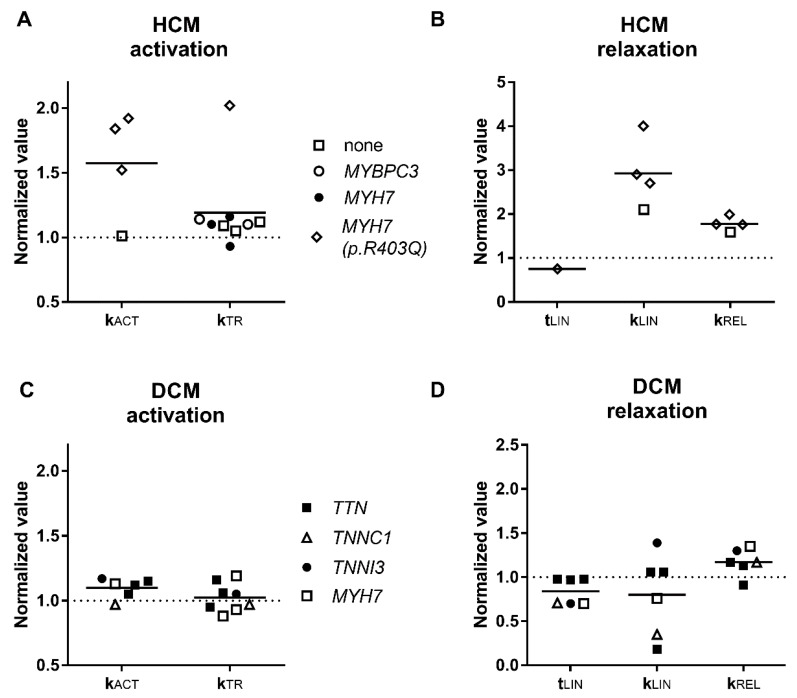
Contractile kinetics parameters. Parameters of activation (**A**,**C**) and relaxation (**B**,**D**) kinetics of HCM and DCM muscle samples. Each data point represents a different experimental group where the symbols indicate genes where mutations were found. All values are normalised to those of donor heart muscle. See also Table 1 and Table 2.

We combined data concerning force development from different papers in Figure 2 and found that the rate of force growth in HCM and DCM samples did not significantly vary from control. Only the samples with mutation in *MYH7* R403Q encoding cardiac *β*-myosin heavy chain, had significantly higher cross bridge turnover rate characterized by the activation and relaxation rate constants [69]. The authors explained it by faster cross-bridge detachment rates. The relaxation kinetics in single R403Q myofibrils was 1.5 times higher than in sarcomere mutation-negative HCM myofibrils and 1.5–2 times higher than in myofibrils from control hearts [71]. Furthermore, it was found 1.6 times higher in the muscle strips of *MYH7* R403Q patients compared to control mutation-negative HCM which showed a positive linear correlation with the slope of slow relaxation phase [71].

Activation kinetics in all studied DCM myofibrils regardless of mutation was not significantly altered. The situation with relaxation was different. Relaxation of DCM samples with truncating mutations in the *TTN* gene was not different from control, while relaxation was faster in all other studied myofibrils which had mutations in the genes of contractile proteins: myosin (*MYH7* E1426K) and troponins (*TNNI3* K36Q and *TNNC1* G159D).

Interestingly, that in the case with RCM disease, myofibril relaxation was slower (Table 2), contributing to diastolic ventricular dysfunction [90].

### 3.3. Elevated Ca^2+^-Sensitivity

**Figure 3:**
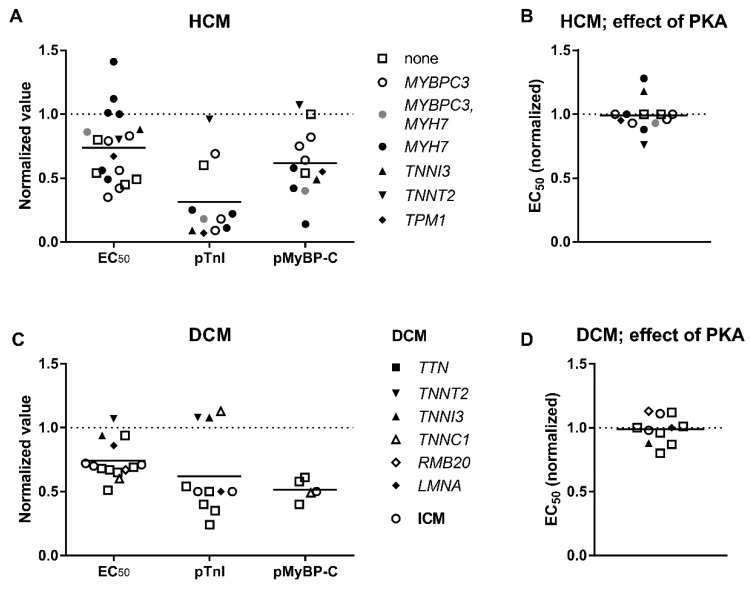
The concentration of Ca^2+^ required for half-maximal contraction (*EC*_50_) and total phosphorylation level of troponin I (pTnI) and MyBP-C (pMyBP-C) of heart tissue samples. The data are from Table 1 and Table 2. The values for HCM (**A**,**B**), DCM (**C**,**D**) are normalized to control donor hearts (dashed line). The effect of PKA treatment on Ca^2+^-sensitivity of heart muscles from HCM (**B**) and DCM (**D**) patients. The *EC*_50_ values are normalized to the *EC*_50_ of normal donor heart samples. PKA eliminates or at least lessens the difference between cardiomyopathic and donor hearts. Each data point represents a different experimental group where the symbols indicate genes where mutations were found.

The Ca^2+^ sensitivity of DCM and HCM patient heart muscle is usually higher (see Figure 3A,C; EC_50 (HCM)_/EC_50 (Donor)_ = EC_50 (DCM)_/EC_50 (Donor)_ = 0.74) than in muscle of a healthy donor. There is a perception that the difference in Ca^2+^-sensitivity is mainly due to TnI dephosphorylation in hearts with cardiomyopathy (Figure 3A,C; 69% and 31% lower in HCM and DCM, respectively) and not because of mutations. In normal donor hearts TnI is highly phosphorylated (0.8–2 mol Pi/mol TnI) and exists mostly in double phosphorylated and single phosphorylated forms. Phosphorylation level is reduced in cardiomyopathies (0.2–1.3 mol Pi/mol TnI) [68,78,84,85,93,94]. But what is true in general may be different for some specific cases. For example, phosphorylation of TnI was found rather high in a number of DCM samples where a mutation was found as the cause of the disease: *TNNI3* p.98trunc and *TNNT2* p.K217del (~2 mol Pi/mol TnI) [78], *ACTC* E99K (HCM mutation, 1.61 mol Pi/mol TnI) [95], *TNNC1* G159D (1.53 mol Pi/mol TnI) [81], and *TNNT2* K280N (1.4–1.6 mol Pi/mol TnI). It may be the case that idiopathic DCM hearts with found mutations may have normal phosphorylation of TnI, whereas hearts without mutation rather have a reduced phosphorylation level. Alongside with TnI, MyBP-C and myosin light chain 2 (or regulatory) phosphorylation also decreased by 1.5–2.5 folds in hearts with cardiomyopathies [67,73,96,97,98]. Phosphorylation of MyBP-C may be responsible for about half of the change in *EC*_50_ at low sarcomere length, but not at high sarcomere length, and is an important factor in length-dependent activation [12].

To discriminate the effect of mutation from the secondary effects caused by dephosphorylation of TnI and MyBP-C, in some experiments skinned muscle samples were treated with protein kinase A (PKA). After PKA treatment, TnI and MyBP-C became phosphorylated to the levels of donor hearts and the difference in *EC*_50_ between a donor heart and heart with cardiomyopathy was ether fully eliminated or substantially reduced (Figure 3B,D). Little difference in the phosphorylation level could be responsible for the variance seen between some patient samples and healthy subject samples.

### 3.4. Uncoupling of TnI Phosphorylation from the Changes in Ca^2+^-Sensitivity

It is well known that phosphorylation of TnI in a normal heart decreases myofilament Ca^2+^-sensitivity. A case has been made that loss of this functional modulation of Ca^2+^-sensitivity through phosphorylation of TnI may play a pivotal role in HCM and DCM development and progression. The experiments using reconstituted thin filamnets in the *in vitro* motility assay provide a strong support that blunted response is one of the common abnormalities seen for both DCM [76,99,100] and HCM mutations [101,102]. It is interesting, that the effect was observed even if pathogenic mutations were not in thin filaments but in other sarcomeric proteins (myosin and MyBP-C) which were not present in the *in vitro* test system [76,101]. Unfortunately, the nature of this blunted response to TnI phosphorylation has not yet been identified.

On the level of myofibrils there is no robust support that such uncoupling exists. The uncoupling was found in myofibrils prepared with the use of animal models of DCM [103,104] where mutations E361G in actin and G159D in troponin C uncoupled myofibrillar Ca^2+^-sensitivity changes from TnI phosphorylation.

**Figure 4:**
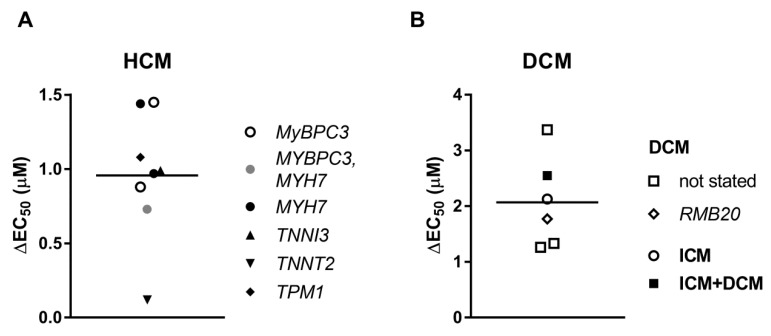
An increase in *EC*_50_ after phosphorylation with protein kinase A (PKA) for HCM (**A**) and DCM (**B**) muscle samples. Each data point represents a different experimental group where the symbols indicate genes where mutations were found. See also Table 1 and Table 2.

The evidence obtained from the *in vitro* experiments with isolated proteins and the studies with the use of animal models [105] is not sufficient. Several studies indicate that uncoupling maybe not an intrinsic property of human HCM [67,73] (Figure 4A). Treatment with PKA decreased Ca^2+^-sensitivity of human cardiomyocytes with HCM mutations in *MYBPC3* and *MYH7*, as well as with mutations in thin filament proteins: *TNNI* R145W and *TNNT2* K280N. The last mutation was identified earlier as an uncoupling mutation [105]. The results plotted on Figure 4 suggest that treatment with PKA has significantly greater effect on Ca^2+^-sensitivity in DCM samples (DCM Δ*EC*_50_ = 1.93 µM; HCM Δ*EC*_50_ = 0.96 µM).

### 3.5. Length Dependent Activation

A length dependent activation can be specified as a contribution of a several components. At first, the maximal force is a function of sarcomere length. Secondly, an increase in sarcomere length considerably shifts the force-[Ca^2+^] curve to the left to lower concentrations of Ca^2+^. The increase in Ca^2+^-sensitivity, in turn, will enhance the rate of contractility. Additionally, the viscoelastic properties also significantly contribute to the total force production. Neither rate of force development (*k*_ACT_, *k*_TR_) [106] nor relaxation kinetics constants (*t*_LIN_, *k*_LIN_, *k*_REL_) are sarcomere length dependent [76].

**Figure 5:**
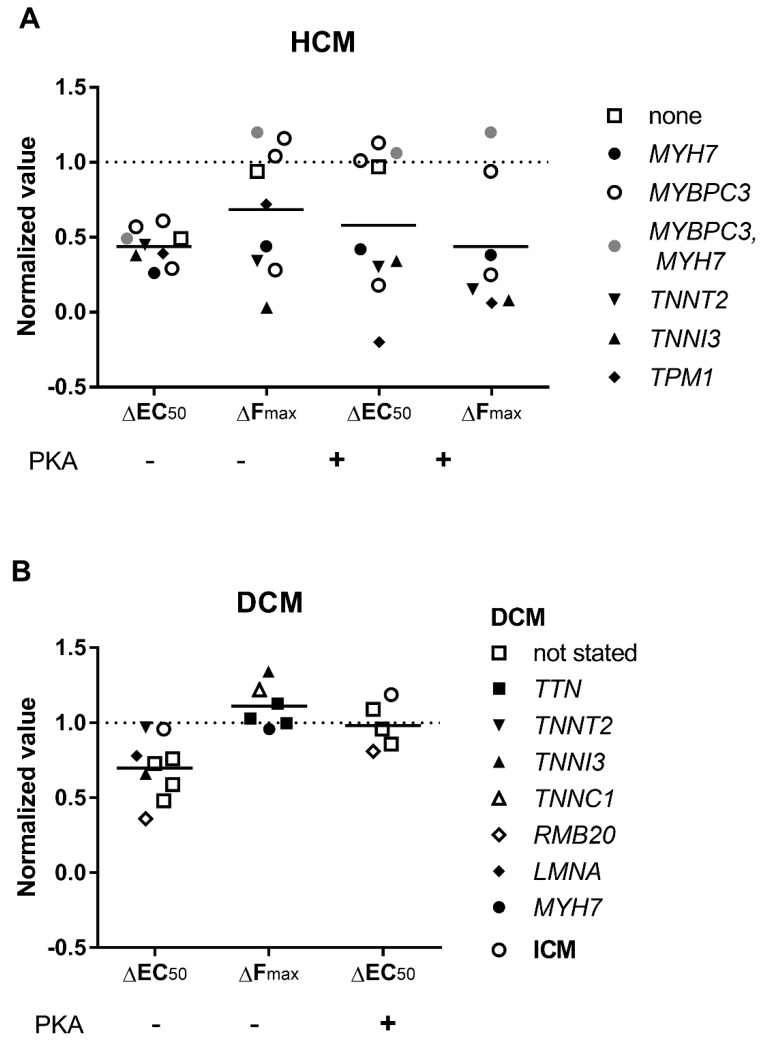
Length dependent activation in cardiac muscle. An increase in sarcomere length decreases *EC*_50_ and increases *F*_max_. Each data point represents a different experimental group where the symbols indicate genes where mutations were found. The values for HCM (**A**) and DCM (**B**) are normalized to control donor hearts (dashed line). The data is from Table 1 and Table 2.

It has been shown that length dependent activation was decreased in HCM samples (Figure 5A). The decrease in *EC*_50_ for Ca^2+^ following sarcomere length increase was reduced in HCM samples by about 56%. Furthermore, the length dependence of force was decreased by 32%. Admittedly, it is rather unusual that length dependent increase of force was fully diminished in patients with mutations in the genes of troponins *TNNI3* and *TNNT2*. In DCM muscles, length dependent activation was less affected: the change in Δ*EC*_50_ decreased by 30% and the force was unchanged (Figure 5B).

There are a number of facts suggesting that the length dependent shift in Ca^2+^-sensitivity was blunted in HCM samples due to the low phosphorylation status of the proteins potentially capable to modulate Ca^2+^-sensitivity: TnI and MyBP-C. Protein kinase A treatment fully restored length dependent activation to a normal level in half of the studies (Figure 5A). A decrease in levels of TnI phosphorylation correlates with a decrease of length dependent activation in DCM samples [48,64] (Figure 5B). MyBP-C phosphorylation (at Ser 275, 284, and 304) was also implied as an important factor capable to modify Ca^2+^-sensitivity and length dependence activation [77]. In this connection we would like to note the fact that HCM samples with truncations and missense mutations in the *MYBPC3* gene show haploinsufficiency of MyBP-C. MyBP-C protein level in these samples was lower by about 30% [66,107,108]. The putative truncated MyBP-C variants were not detected in cardiac tissue samples by Western blot [109]. It suggests protein expression is very low or such mutant proteins undergo degradation in the ubiquitin-proteasome proteolytic pathway [110]. Such overloading of the ubiquitin-proteasome is one of the putative factors to initiate the disease.

### 3.6. Passive Stiffness

**Figure 6:**
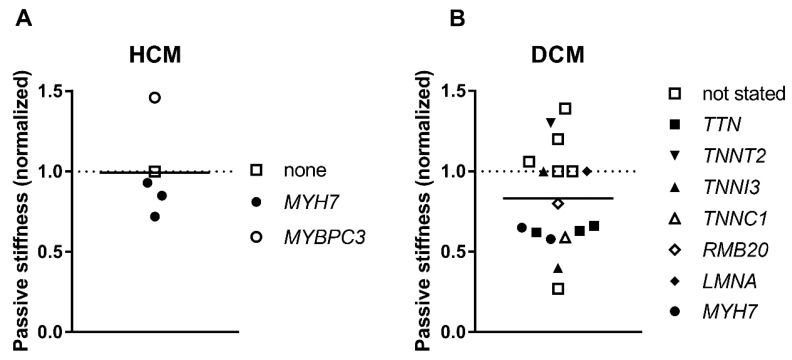
Passive stiffness. Passive stiffness was evaluated as a Young’s modulus or as a passive tension generated by muscle at stretch. Each data point represents a different experimental group where the symbols indicate genes where mutations were found. The values for HCM (**A**) and DCM (**B**) are normalized to control donor hearts (dashed line). The data is from Table 1 and Table 2.

Titin is the major contributor to myofibril elasticity, and it is the main force component during diastole accumulating the potential energy of stretch and discharging it during systole. It was shown that passive myofibrillar stiffness was not substantially changed in HCM (Figure 6A, Table 1). Dilated cardiomyopathy-linked mutations in *TTN* and other sarcomeric proteins as well as with no found mutations decrease titin elasticity characterized by a decrease in myofibrillar stiffness by about 26% (Figure 6B, Table 2) [76,111,112]. The puzzling thing here is that putative truncated variants were not found in myofibril sarcomeres [76,113]. In some cases, the decrease in passive stiffness was, to some extent, explained by altered titin isoform expression [111,112]. However, in many cases there was no correlation between passive stiffness and differential titin isoform expression (Table 2). The slack or resting sarcomere length of DCM myocytes or myofibrils was matching to control [76,80,85], with a difference only found with *RMB20* E913K mutation, where expression of the highly compliant N2BA titin isoform was several times higher [80]. The resting sarcomere length in HCM myocytes was similar to control [69] or slightly shorter in some cases [65].

## 4. Conclusions

In this review we tried to analyze data from different studies to show some main correlations between the mutations in the sarcomere proteins or their modification associated with different types of cardiomyopathies and the contractile properties of heart muscle from HCM and DCM patients.

Data from Table 1 shows that the inability of HCM cardiac myofibrils to generate the same force as controls is one of the essential differences between HCM and DCM muscles (Figure 1A,C). As defined, maximal force depends on the number of force-generating myosin heads and the rates of myosin cross-bridge attachments and detachments [114]. It is assumed that the increase of cross-bridge detachment rate leads to the detrimental increase in the energetic cost of contraction that may be the underlying mechanism of HCM. This hypothesis requires further research by testing contractility and the efficiency of ATP consumption in human cardiac muscles with different HCM mutations. The decrease of force was not observed for DCM myofibrils, but the rate of relaxation was also higher in samples with mutations in the contractile proteins (Figure 2D) [76].

The diastolic intracellular concentration of Ca^2+^ is about 0.2 μM and it increases to about 1.5 μM in systole [115,116,117]. An impaired contractility in HCM may be compensated by increased Ca^2+^-sensitivity regulated via dephosphorylation of TnI. Although an increase in Ca^2+^-sensitivity is the main feature of all cardiomyopathies and heart failure, dephosphorylation of TnI was the major factor responsible for this increase (Figure 3). Therefore, it is essential here to differentiate the direct effect of mutations in proteins on myofibril contractility from other secondary effects. It was proposed that high myofilament Ca^2+^-sensitivity slows myofibril relaxation in diastole [8,118,119]. Furthermore, it is interesting to investigate if the coupling between changes in TnI phosphorylation and the Ca^2+^-sensitivity of force in HCM and DCM myofibrils is reduced, particularly if a mutation was found in thin filaments. Additionally, titin mechanical properties are believed to be involved to the work output characteristic of myocardium. The decrease of titin originated stiffnes can cause systolic dysfunction in DCM heart muscles [76,112].

## Figures and Tables

**Table 1:** Contractile characteristics of hypertrophic cardiomyopathy heart muscle.

	Mutation, Gene Expression and Mutated (mut) Allele Expression					Phosphorylation Level (Cardiomyopathy/Healthy)			Titin Isoforms and Passive Stiffness (Cardiomyopathy/Healthy)		Maximal Force and Myofibril Density (Cardiomyopathy/Healthy)			Contractile Kinetics Parameters (Cardiomyopathy/Healthy)					Ca^2^-Sensitivity of Force (Cardiomyopathy/Healthy)			Length Dependent Activation Changes upon Stretch (Cardiomyopathy/Healthy)			Effect PKA on EC_50_ (in µM)		Patient Information (Sex, Age and Number of Patients)			
				Expression							Myocyte		Myofibril	Activation		Relaxation					+PKA			+PKA						
Diagnosis	Gene with Mutation	Mutation and Type: Truncated (T) or Not (NT)		Gene	Mut Allele	TnI	MyBP-C	LC2	N2AB/N2B	Passive Stiffness	*F*_max_	Myofibril Density	*F*_max_	*k*_ACT_	*k*_TR_	*t*_LIN_	*k*_LIN_	*k*_REL_	*EC*_50_	*n*_H_	*EC*_50_	Δ*EC*_50_	Δ*F*	Δ*EC*_50_	Δ*F*	ΔEC_50_	Sex M/F	Age	N	Refs
HCM	*MYBPC3*	g.T2604A+C del at 2605	T	0.64 *	0.34 *						0.64	1			1.14				0.42								M	42	1	[65,66]
HCM	*MYBPC3*		T			0.18	0.75				0.85 *								0.79 *		0.96	0.57 *	1.16	1.01	0.94	0.88 *	M/F	22–69	17	[67]
HCM	*MYBPC3*		T	0.67		0.69 *	0.82			1.46	0.71 *								0.83 *	0.84 *	1	0.61 *	1.04	1.13			M/F	22–69	17	[68]
HCM	*MYBPC3*		T/NT								0.68 *	0.69 *	0.85 *							1	1						MF	32–69	28	[57]
HCM	*MYBPC3*	p.R502W	NT	0.8 *							0.75				1.10				0.35								M	23	1	[65]
HCM	*MYBPC3*		NT			0.09	0.64				0.44 *								0.56 *		0.93	0.29 *	0.28 *	0.18 *	0.25 *	1.45 *	M/F	31–51	4	[67]
HCM	*MYH7*	p.R719Q	NT								0.79				1.16				1.01								F	27	1	[65]
HCM	*MYH7*		NT			0.22	0.58				0.54 *								0.56 *		0.88 *	0.26 *	0.44 *	0.42 *	0.38 *	0.97 *	M/F	25–61	4–6	[67]
fHCM	*MYH7*	p.R403Q	NT		0.35 *					0.93	0.3		0.56 *	1.92 *	2.02 *	0.75 *	4 *	1.76 *									M	24	1	[69,70,71]
fHCM	*MYH7*	p.R403Q	NT		0.5						0.6		0.83	1.84			2.9	1.77									M	35	1	[69,70,71]
fHCM	*MYH7*	p.R403Q	NT		0.45						0.6		0.91	1.52			2.7	1.99									F	39	1	[69,70,71]
fHCM	*MYH7*	p.R723G	NT		0.7						0.54 *								1.41 *								M	38, 55	2	[72]
fHCM	*MYH7*	p.R723G	NT		0.68	0.11 *	0.42 *	0.42 *		0.72 *	0.69 *	0.74 *			0.93				1		1.28 *					1.44 *	M/F	53, 55	2	[73,74]
fHCM	*MYH7*	p.A200V	NT		0.53						0.48 *								1.12 *								F	19	1	[72,75]
fHCM	*MYH7*		NT								0.39 *	0.79 *	0.65 *								1						M/F	19–61	11	[57]
fHCM	*TPM1*	p.I284V	NT								0.40 *	0.70 *	0.62 *														M	65	1	[57]
HCM	*TPM1*	p.M281T	NT			0.07	0.55				0.48 *								0.67 *		0.95	0.39 *	0.72	−0.2 *	0.06 *	1.08 *	M	65	1	[67]
HCM	*TNNT2*	p.K280N	NT			0.96	1.07				0.70								0.80 *		0.76 *	0.45 *	0.34	0.30 *	0.15 *	0.12	M	26	1	[67]
HCM	*TNNT2*	p.K280N	NT								0.56	0.78 *	0.73 *														M	26	2	[57]
HCM	*TNNI3*	p.R145W	NT								0.55 *	0.58 *	0.84														M	46, 66	2	[57]
HCM	*TNNI3*	p.R145W	NT			0.09	0.49				0.29								0.88 *		1.18 *	0.38 *	0.03 *	0.34 *	0.08 *	0.99 *	M	46, 66	2	[67]
HCM	*MYH7*(2) *MYBPC3*(1)		NT			0.25 *	0.14 *	0.91	0.70	0.85	0.61 *	1			1.1 *				0.49 *	1.26							M/F	23–61	6	[65,70]
HCM	none					0.18	0.4				0.81 *								0.86 *		0.93	0.49 *	1.2	1.06 *	1.2	0.73	M/F	46–75	3–7	[67]
HCM	none						1				0.71 *	0.92 *	0.78 *								1						M/F	35–75	3–14	[57]
HCM	none					0.60 *	0.54 *			1	0.75 *								0.8 *	0.81 *	1	0.49 *	0.94	0.97			M/F	46–75	11	[68]
HCM	none										1.0		0.84	1.01			2.1	1.59									M/F	35–72	9	[71]
HCM	none										0.61				1.09				0.54								M	59	1	[65]
HCM	none										0.35				1.05				0.45								F	61	1	[65]
HCM	none										0.42				1.12				0.49								M	58	1	[65]

The value changes were normalized to healthy control hearts (Cardiomyopathy/Healthy) if not otherwise specified. The parameters of length dependent activation are shown as a difference in *EC*_50_ and *F*_max_ between long and short sarcomere lengths. Passive stiffness was evaluated as a Young’s modulus or as a passive tension generated by muscle at stretch. In some cases, approximate values are given. * *p* < 0.05. HCM—hypertrophic cardiomyopathy, fHCM—familial HCM, PKA—protein kinase A. The table cells are highlighted according to the value changes compared to control: yellow—no changes, green—the value decreased, blue—the value increased.

**Table 2:** Contractile characteristics of dilated and other cardiomyopathy heart muscles.

	Mutation, Gene Expression and Mutated (mut) Allele Expression					Phosphorylation Level (Cardiomyopathy/Healthy)			Titin Isoforms and Passive Stiffness (Cardiomyopathy/Healthy		Maximal Force and Myofibril Density (Cardiomyopathy/Healthy)			Contractile Kinetics Parameters (Cardiomyopathy/Healthy)					Ca^2^-Sensitivity of Force (Cardiomyopathy/Healthy)			Length Dependent Activation Changes upon Stretch (Cardiomyopathy/Healthy)			Effect PKA on EC_50_ (in µM)	Patient Information (Sex, Age and Number of Patients)			
				Expression							Myocyte		Myofibril	Activation		Relaxation					+PKA			+PKA					
Diagnosis	Gene with Mutation	Mutation and Type: Truncated (T) or Not (NT)		Gene	Mut Protein	TnI	MyBP-C	LC2	N2AB/ N2B	Passive Stiffness	*F*_max_	Myofibril Density	*F*_max_	*k*_ACT_	*k*_TR_	*t*_LIN_	*k*_LIN_	*k*_REL_	*EC*_50_	*n*_H_	*EC*_50_	Δ*EC*_50_	Δ*F*	Δ*EC*_50_	Δ*EC*_50_	Sex M/F	Age	N	Refs
IDCM	*TTN*	p.(R23464Tfs *41)	T	1.0	0				1.05	0.63 *			1.02	1.05	1.06	0.97	1.06	0.91					1.00			M	22	1	[76]
fDCM	*TTN*	p.(R23464Tfs*41)	T	1.0	0				1.11	0.66 *			0.96	1.12	0.95	0.98	0.18	1.13					1.13			M	37	1	[76]
fDCM	*TTN*	p.(Y18923 *)	T	0.96	0				1.25	0.62 *			1.04	1.15	1.16 *	0.98	1.06	1.17					1.03			F	22	1	[76]
PPCM	*TTN*	p.(K15664Vfs*13)	T						1.85	0.4																F		1	[77]
DCM	*LMNA*	p.(R331Q)	NT			0.5			1.98 *	1	0.64 *	0.63							0.86 *		1	0.78				M/F	45.3	3	[78,79]
fDCM	*RMB20*	p.E913K	NT	0.13					13.89	0.8	0.85								0.67 *	0.58	1.13	0.36		0.81	1.77	M	19	1	[80]
fDCM	*TNNC1*	p.G159D	NT			1.13	0.49	0.13	0.89	0.59 *	1		1.20	0.97	0.97	0.71 *	0.35	1.17	0.60 *	0.59 *			1.22			M	3	1	[76,81]
fDCM	*TNNI3*	p.K36Q	NT						1.0	0.58 *			1.10	1.17	1.05	0.70 *	1.39	1.30 *					1.34			M	15	1	[76]
IDCM	*TNNI3*	p.(R98 *)	T	0.39	0	1.08			1.56	1	0.95								0.94 *		0.88 *	0.66				M	46	1	[78,82]
IDCM	*TNNT2*	p.(K217del)	NT	0.51		1.08			1.64	1.3 *	1.11								1.07			0.97				M	19	1	[78]
fDCM	*MYH7*	p.E1426K	NT						1.08	0.65 *			0.97	1.13	1.19 *	0.70 *	0.76	1.35 *					0.96			M	43	1	[76]
IDCM	not stated					0.54			1.98	1	0.98								0.94		1	0.73				M/F	54.6	5	[78]
DCM	not stated								1.24 *	0.27 *																	51–56	3–4	[83]
DCM	not stated					0.5													0.51	1	0.96				3.37	M	45,59	2	[84]
IDCM	not stated					0.35 *	0.58 *		1.62 *	1	0.94								0.69 *		1.12	0.76		1.09				8	[85]
IDCM	not stated					0.4 *	0.4 *	1.1			1.18				0.88				0.67	1.33	0.8				1.26	M/F	52–62	7	[86]
IDCM	not stated								1.78	1.2	1.34								0.68 *	0.81	0.87	0.59		0.86	1.33			3	[80]
IDCM	not stated								1.14																	F	41–57	6	[87]
PPCM	not stated					0.24 *	0.61 *		1.58	1.39 *	0.84								0.65 *		1.01	0.48 *		0.96		F		6	[85]
ICM	not stated										0.95				0.93				0.71 *	0.83 *								3	[88]
ICM	not stated					0.5													0.72 *		0.98				2.13	M/F	41–65	7	[84]
ICM	not stated					0.5	0.5 *		0.83	1	1.04								0.70 *		1.11	0.96		1.19				4	[85]
ICM (7) DCM (2)	not stated					0.5		0.57 *		1.06	1.0								0.66 *	0.99	0.98				2.55 *	M/F	41–65	10	[84]
HF	not stated					0.33 *	0.65 *	1		0.53 *	0.53 *				0.96				0.63 *	0.87	0.98					M/F	42–57	4	[89]
IRCM	not stated												0.84			1.20 *	0.05 *	0.52 *								F	66	1	[90]
IRCM	not stated												1.16			1.92 *	0.07 *	0.33 *								M	21	1	[90]
IDCM (9) NCC (2)	not stated					0.27 *			0.4–2.8	0.7 *	0.64 *											0.67 *		1			<18	11	[58]

The value changes were normalized to healthy control hearts (Cardiomyopathy/Healthy) if not otherwise specified. The parameters of length dependent activation are shown as a difference in *EC*_50_ and *F*_max_ between long and short sarcomere length. Passive stiffness was evaluated as a Young’s modulus or as a passive tension generated by muscle at stretch. In some cases, approximate values are given. * *p* < 0.05. PPCM—peripartum cardiomyopathy, ICM—ischemic cardiomyopathy, RCM—restrictive cardiomyopathy, HF—heart failure, IDCM—idiopathic DCM, fDCM-familial DCM, IRCM—idiopathic restrictive cardiomyopathy. The table cells are highlighted according to the value changes compared to control: yellow—no changes, green—the value decreased, blue—the value increased.
